# The Value of Lymph Node Dissection in Patients With Node-Positive Upper Urinary Tract Urothelial Cancer: A Retrospective Cohort Study

**DOI:** 10.3389/fonc.2022.889144

**Published:** 2022-06-16

**Authors:** Hao-ran Xia, Shu-guang Li, Xing-quan Zhai, Min Liu, Xiao-xiao Guo, Jian-ye Wang

**Affiliations:** ^1^ Department of urology, Beijing Hospital, National Center of Gerontology; Institute of Geriatric Medicine, Chinese Academy of Medical Science, Beijing, China; ^2^ Department of Urology, Zoucheng People`s Hospital, Zoucheng, China; ^3^ Department of Urology, Beijing Friendship Hospital, Capital Medical University, Beijing, China

**Keywords:** upper urinary tract urothelial cancer, lymphadenectomy, removed lymph nodes, positive lymph nodes, positive lymph node density

## Abstract

**Purpose:**

The value of lymphadenectomy during radical nephroureterectomy (RNU) remains unclear. This study aimed to determine the effects of the removed lymph node (RLN) counts, positive lymph node (pLN) counts, and pLN density (pLND) on survival outcomes in patients with node-positive upper urinary tract urothelial cancer (UTUC).

**Methods:**

A total of 306 patients with node-positive UTUC in the Surveillance, Epidemiology, and End Results database between 2004 and 2016 were identified. Multivariable Cox regression analyses were used to evaluate the effect of RLN counts, pLN counts, and pLND on survival outcomes. The maximally selected rank statistics were used to determine the most informative cutoff value for pLND on survival outcomes.

**Results:**

The RLN counts or pLN counts were not associated with survival outcomes, whereas higher pLND was associated with lower cancer-specific survival (CSS) and overall survival (OS) [hazard ratio (HR) 1.75, *P* = 0.014 and HR 1.62, *P* = 0.036, respectively]. The most informative cutoff value for pLND in relation to survival was 27%. Patients with pLND ≥27% had worse 5-year survival rates than those with pLND <27% (52.9% *vs.* 75.9% for CSS and 18.7% *vs.* 34.2% for OS, each *P* < 0.05). Furthermore, the multivariable Cox regression model with pLND could predict 5-year CSS (AUC 0.732 *vs.* 0.647) or OS (AUC 0.704 *vs.* 0.621) more accurately than the model without pLND.

**Conclusions:**

For patients with node-positive UTUC, more lymph nodes removed do not offer a better therapeutic effect. However, pLND provides additional prognostic value.

## Introduction

Regional lymph node metastasis (LNM) accounts for approximately 30%–40% of patients with muscle-invasive urinary tract urothelial cancer (UTUC) (1), and it is an independent predictor for worse survival outcomes (2). The latest guidelines indicate that lymphadenectomy performed simultaneously with radical nephroureterectomy (RNU) allows for optimal tumor staging; however, its curative role remains controversial (3). The existing results are largely derived from studies that compared the survival between patients without pathological nodal assessment (pNx disease) and those with pathologically confirmed node-negative (pN0 disease). It is of note that in pN0 versus pNx patients, the survival advantage of the former could be driven by the Will Rogers effect. Therefore, it is necessary to test the effects of lymph node dissection in a properly selected population, in whom the lymph node staging is pathologically confirmed. In this study, based on the Surveillance, Epidemiology, and End Results (SEER) database, we evaluated the effects of the removed lymph node (RLN) counts, positive lymph node (pLN) counts, and positive lymph node density (pLND) on the survival outcomes in patients with node-positive UTUC which was confirmed by the pathological results of lymphadenectomy.

## Patients and Methods

### Patient Cohort

**Figure 1:**
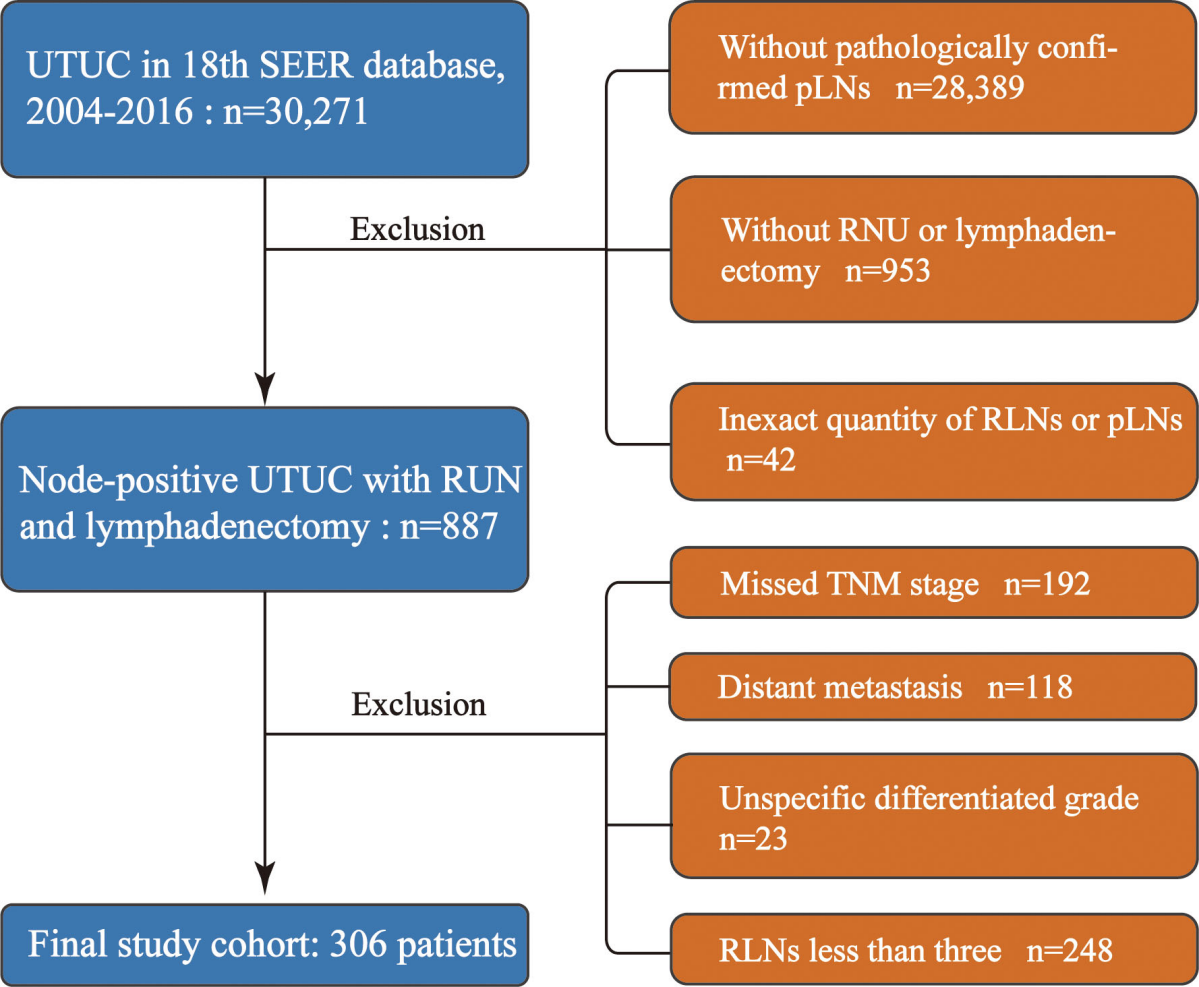
Study cohort selection. UTUC, upper urinary tract urothelial cancer; RNU, radical nephroureterectomy; RLNs, removed lymph nodes; pLNs, positive lymph nodes.

Our study was granted an exemption from the Ethics Review Board because the Surveillance, Epidemiology, and End Results Program collects data from population-based cancer registries with anonymous information and the database is public. The information of the patients was retrospectively collected from the SEER 18th database from 2004 to 2016. A total of 30,271 patients with histologically confirmed UTUC were primarily screened. The inclusion criteria were as follows: 1) patients with UTUC as the first primary malignancy, 2) patients with positive lymph nodes, and 3) patients who underwent RNU and lymphadenectomy. We excluded those with organ metastasis and missing data regarding TNM stage, tumor size, and the exact quantity of RLNs or pLNs. Furthermore, patients with <3 RLNs were excluded to make the median of LN yield in present study reach the LN yield in previous studies which conducted template lymphadenectomy (4, 5). The patient inclusion and exclusion diagrams are depicted in **Figure 1**.

### Definition of Variables for Analyses

Tumor stages were described as T2, T3, and T4. In SEER, the tumor grades were classified as well-differentiated (grade I), moderately differentiated (grade II), poorly differentiated (grade III), and undifferentiated (grade IV). In this study, pathological grades were divided into low grade (LG) for SEER grades I–II and high grade (HG) for SEER grades III–IV. Tumor size was classified as <2 and ≥2 cm. The definition of pLND is the number of positive nodes divided by the total number of resected nodes. In the present study, pLND was calculated by dividing the pLN counts by the RLN counts. The major endpoints of interest were UTUC-specific survival (CSS) and overall survival (OS).

### Statistical Analysis

The median [interquartile range (IQR)] was reported for continuous variables. The frequency (proportion) was reported for categorical variables. Univariable analysis was performed before multivariable Cox regression. Any variable having a significant univariate test at an arbitrary level is selected as a candidate for the multivariate analysis. We based this on the *P*-value cutoff point of 0.5. Multivariable Cox regression analyses were used to explore the relationship of RLN counts, pLN counts, and pLND with OS and CSS. Furthermore, a multivariable Cox regression model with a penalized spline was used to examine the relationship between pLND as a continuously coded variable with CSS (6). The most informative cutoff value of pLND was calculated using maximally selected rank statistics based on log-rank analysis and used to stratify the patients into two groups. The area under the receiver operating characteristic curve (AUC) was used to evaluate the predictive ability of clinicopathological parameters for 5-year survival. All tests were two-sided and *P <*0.05 was considered statistically significant. All analyses were performed using R software (version 3.6.1).

## Results

### Baseline Patient Characteristics

As shown in **Table 1**, of the 306 node-positive UTUC patients, the sex distribution was 55.6% versus 44.4% for men and women, respectively. A total of 45.1% of the tumors were found on the left side. Of all the tumors, 27.8% and 72.2% were located in the ureter and renal pelvis, respectively. The median age of the patients was 71 years (IQR 62–79 years), 85.6% of the patients have T3 or T4 disease, and a total of 95.5% of all tumors were pathological high grade. Among all individuals, 7.5% and 52.5% received adjuvant radiotherapy and adjuvant chemotherapy, respectively. The median number of RLNs and pLNs and the pLND were 8 (IQR 6–14), 3 (IQR 1–5), and 33.3% (IQR 16.9%–66.7%), respectively. The median follow-up period was 16.5 (IQR 8.0–29.0 months).

**Table 1:** Baseline demographic and clinicopathologic characteristics of the patients.

Characteristic	Patients (*n* = 306)
**Age, years**	71 [62, 79]
**Year of diagnosis**	
2000–2005	61 (19.9)
2006–2010	96 (31.4)
2011–2016	149 (48.7)
**Sex**	
Male	170 (55.6)
Female	136 (44.4)
**Race**	
Caucasian	254 (83.0)
African	35 (11.4)
Other	17 (5.6)
**Tumor site**	
Renal pelvis	221 (72.2)
Ureter	85 (27.8)
**Laterality**	
Left	138 (45.1)
Right	168 (54.9)
**Tumor size**	
<2 cm	22 (7.2%)
≥2 cm	284 (92.8%)
**T stage**	
T2	44 (14.4)
T3	153 (50.0)
T4	109 (35.6)
**Pathological grade**	
Low grade	28 (4.5)
High grade	278 (95.5)
**RLNs**	8 [6, 14]
**pLNs**	3 [1, 5]
**pLND, %**	33.3 [16.9, 66.7]
**Adjuvant radiotherapy**	
Yes	23 (7.5)
No	283 (92.5)
**Adjuvant chemotherapy**	
Yes	161 (52.5)
No	145 (47.5)
**Follow-up, months**	16.5 [8, 29]

Data are presented as n (%) or median [IQR]. RLNs, removed lymph nodes; pLNs, positive lymph nodes; pLND, positive lymph node density; IQR, interquartile range.

### Cox Regression Analyses and pLND Stratification

As shown in **Supplementary Table 1**, all variables met the criterion for the candidate of multivariate analysis. In multivariable Cox regression analysis, **Table 2** shows that older age [hazard ratio (HR) 1.01], tumor size ≥2 cm (HR 2.06), T4 stage (HR 3.97), without adjuvant chemotherapy (HR 3.61), and higher pLND (HR 1.62) were associated with poor OS (all *P* < 0.05). However, pathological grades, the number of RLNs, adjuvant radiotherapy, and pLNs were not associated with OS (each *P* > 0.05). As for CSS, tumor size ≥2 cm (HR 2.26), T3 and T4 stages (HR 2.08 and 3.84, respectively), without adjuvant chemotherapy (HR 2.17), and higher pLND (HR 1.75) were independent predictors of lower CSS (**Table 2**), whereas pathological grades, the number of RLNs, and pLNs were not associated with CSS (each *P* > 0.05). The results of the multivariable Cox regression analyses of RLNs or pLNs and covariates for OS and CSS are shown in **Supplementary Tables 2** and **3**.

**Table 2:** Multivariable Cox regression analyses of pLND and covariables for overall survival and cancer-specific survival outcomes.

	CSS		OS	
	HR (95% CI)	*P*-value	HR (95% CI)	*P*-value
**Age**	1.00 (0.98–1.03)	0.749	1.01 (1.00–1.03)	0.048
**Sex**				
Male	Ref.		Ref.	
Female	1.13 (0.82–1.57)	0.455	0.76 (0.47–1.21)	0.246
**Race**				
Caucasian			Ref.	
African	0.78 (0.38–1.60)	0.500	0.48 (0.17–1.33)	0.155
Other	0.84 (0.51–1.37)	0.477	0.87 (0.43–1.77)	0.704
**Tumor site**				
Renal pelvis	Ref.		Ref.	
Ureter	0.67 (0.45–1.00)	0.051	0.62 (0.34–1.13)	0.120
**Laterality**				
Left	Ref.		Ref.	
Right	1.05 (0.76–1.44)	0.771	1.01 (0.64–1.58)	0.979
**Tumor size**				
<2 cm	Ref.		Ref.	
≥2 cm	2.26 (1.05–4.87)	0.038	2.06 (1.11–4.29)	0.012
**T stage**				
T2	Ref.		Ref.	
T3	2.08 (1.13–3.82)	0.018	1.81 (0.78–4.17)	0.167
T4	3.84 (2.04–7.24)	<0.001	3.97 (1.67–9.43)	0.002
**Pathological grade**				
Low grade	Ref.		Ref.	
High grade	2.71 (0.60–12.2)	0.203	2.59 (0.97–6.41)	0.062
**Adjuvant radiotherapy**				
No	Ref.		Ref.	
Yes	0.95 (0.54–1.67)	0.861	1.21 (0.50–2.91)	0.678
**Adjuvant chemotherapy**				
Yes	Ref.		Ref.	
No	2.17 (1.55–3.03)	<0.001	3.61 (2.22–5.86)	<0.001
pLND	1.75 (1.13–2.32)	0.014	1.62 (1.03–2.41)	0.036

HR, hazard ratio; CI, confidence interval; CSS, cancer-specific survival; OS, overall survival; pLND, positive lymph node density.

**Figure 2:**
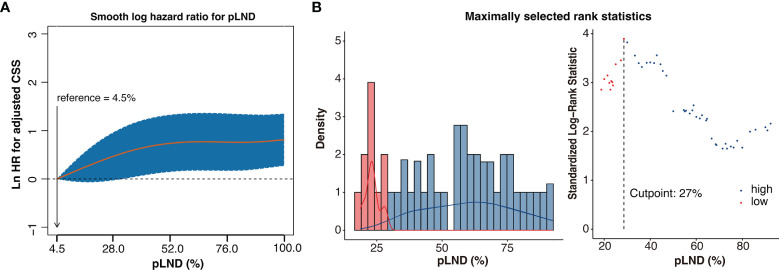
Non-linear-dependent effect of pLND on cancer-specific survival and the scatter plot of maximally selected rank statistics for the most informative cutoff point for pLND. **(A)** The estimated logarithm HR (yellow line) with 95% CI (dash curves) for the association of the pLND with CSS in 306 patients, on the basis of the dfmacox in a smoothHR—the optimal extended Cox-type additive hazard regression adjusted for covariates. pLND was used as the continuous variable, and the effect of pLND on the risk of mortality was modeled using a penalized spline (P-spline) expansion. pLND = 4.5% was used as the reference value for calculating the HR, respectively. Ln HR >0 represents a higher cancer-specific mortality risk. **(B)** The scatter plot of maximally selected rank statistics shows that the cutoff value for pLND is 27%. HR, hazard ratio; pLND, positive lymph node density; CSS, cancer-specific survival.

**Figure 3:**
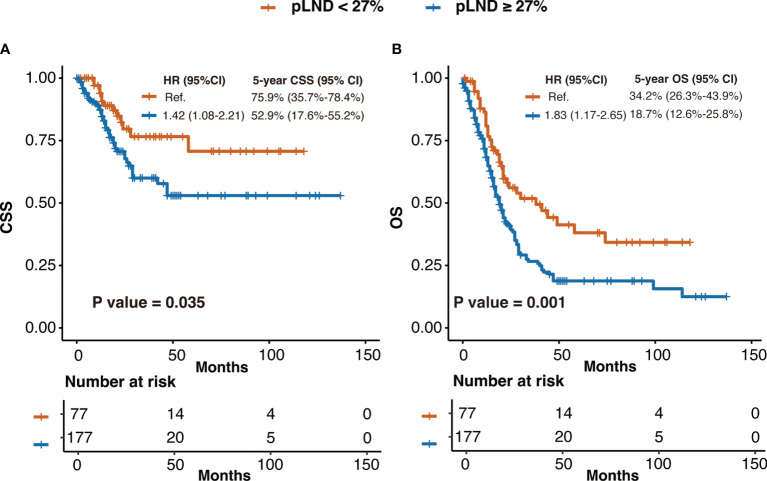
The Kaplan–Meier curves of CSS **(A)** and OS **(B)** after patients were stratified according to 27% pLND. HR, hazard ratio; CI, confidence interval; pLND, positive lymph node density; CSS, cancer-specific survival; OS, overall survival.

As shown in **Figure 2A**, CSS risk increases with pLND; however, the relationship between the variables is “non-linear.” Therefore, we calculated the most informative cutoff value for pLND on CSS. The results of the maximally selected rank statistics suggested that the value was 27.16% (**Figure 2B**). Furthermore, the Kaplan–Meier survival curves show that the 5-year CSS and OS rates for patients with <27% pLND compared with their counterparts with ≥27% pLND were 75.9% versus 52.9% (*P* = 0.035) and 34.2% versus 18.7% (*P* = 0.001), respectively (**Figure 3**).

**Figure 4:**
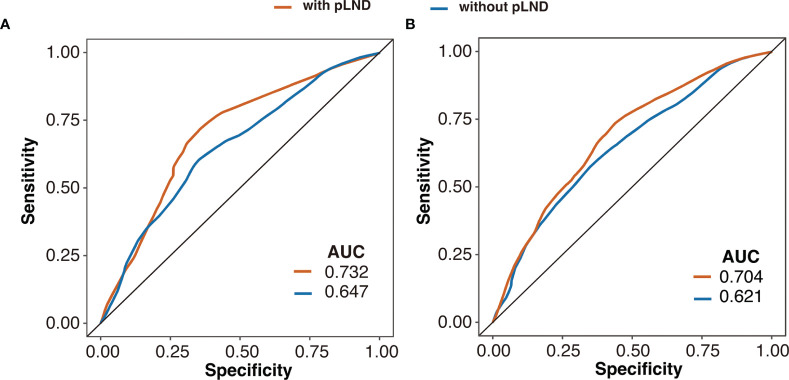
Receiver operating characteristic curves of the 5-year cancer-specific survival **(A)** and overall survival **(B)** based on multivariable Cox proportional hazards model with or without pLND. AUC, area under the curve; pLND, positive lymph node density.

The predictive efficacy of the multivariable Cox proportional hazards model with or without pLND is shown in **Figure 4**. The model with pLND could predict the 5-year CSS or OS more accurately, and the AUC values were 0.732 versus 0.647 and 0.704 versus 0.621 for the model with or without pLND, respectively.

## Discussion

LNM and extranodal extension are powerful predictors of survival outcomes in UTUC (7, 8). It has been recognized that lymphadenectomy performed at the time of RNU allows for optimal tumor staging; however, its curative role remains controversial (9, 10). This population-based study aimed to add evidence regarding the value of lymphadenectomy for patients with node-positive UTUC.

Most of the patients in the present study harbored a tumor with advanced clinicopathological features, such as tumor size ≥2 cm, T3 or T4 stage, and pathologically high grade, which represent the real clinical scenario—the risk of LN metastasis increases with advancing tumor stage (10) and lymphadenectomy was always performed for high-risk UTUC but not for low-risk disease (11, 12). The results showed that bigger tumor size and higher tumor stage were associated with worse survival outcomes. However, pathological grade was not a survival predictor. The primary reason could be that patients with low-grade tumors are too few to reach a statistical difference.

The data reported here suggest that RLN counts had a little impact on the survival of patients with node-positive UTUC. A few studies, as yet, have evaluated the effect of lymphadenectomy on the survival outcomes of UTUC, and their conclusions, however, are different. Inokuchi et al. retrospectively examined the outcomes in 2,032 patients who underwent RNU for UTUC. Of these, 991 underwent concomitant lymphadenectomy. The authors found no difference in OS or CSS between patients who underwent lymphadenectomy and those who did not. Furthermore, higher RLN counts did not confer better survival outcomes (13). In contrast, Abe et al. retrospectively reviewed 312 patients who underwent RNU, and 166 of these patients underwent lymphadenectomy. The 5-year CSS was 88.4% for the pN0 group and 64.7% for the pNx group, and a significant difference in survival was observed between the two groups (14). The present study included only node-positive patients and found that RLN counts were not associated with CSS or OS in these individuals. A possible reason for the discrepancy in the conclusion of the studies could be attributed to the Will Rogers effect, i.e., in those studies comparing the survival outcomes of pNx patients versus pN0 patients, the latter were pathologically confirmed node-negative, whereas a substantial part of pNx patients probably harbored positive LNs, therefore resulting in the worse survival outcomes in pNx individuals.

The present study suggested that pLN counts had no prognostic value for patients with node-positive UTUC. The result is in accordance with that reported in the study by Bolenz et al. They reviewed the data of 135 patients with node-positive UTUC treated with RNU, investigated the risk factors associated with clinical outcomes, and found that pLN count was not associated with CSS or cancer recurrence (15). Moreover, Fajkovic et al. performed a retrospective review of 222 node-positive UTUC patients to assess the prognostic value of extranodal extension and other lymph node parameters after RNU (8). They found that pLN count was not associated with recurrence or cancer-specific mortality. However, in a previously mentioned SEER-based study by Zareba et al. (12), the authors reported that a higher pLN count was associated with lower OS in 771 node-positive UTUC patients. One possible explanation for the contradictory result between the studies is that the inconsistent lymphadenectomy pattern limited the accuracy of pLN counts, resulting in the discrepancy of conclusions. Indeed, for a portion of patients with low LN yield, LN dissection during RNU would be considered a staging procedure with selective removal of suspicious LNs (“node plucking”) without interest in extended lymphadenectomy, which would result in pLN yield being less than the actual number. On the other hand, Matin et al. (16) added to the work of Kondo et al. (5) in predicting the patterns of LNM according to the laterality and location of the tumor. Nevertheless, the extent of lymphadenectomy during RNU is largely determined by different surgeons.

pLND has been established as an important prognostic factor in patients with other genitourinary carcinomas, such as prostate cancer and bladder cancer (17–19). In the current study, we found that a higher pLND was associated with poorer outcomes in patients with node-positive UTUC, and the relationship between them was non-linear. Therefore, there may be an optimal informative cutoff value for pLND that can be used for further risk stratification in these patients. For this study, the best pLND cutoff for the prediction of CSS was 27.16%, and patients with <27% pLND had a better CSS and OS than those with ≥27% pLND. Furthermore, the multivariable Cox regression model that included pLND showed a higher accuracy for 5-year survival rate prediction. Our findings validated the results reported by Bolenz et al., in which pLND ≥30% was associated with a greater 5-year recurrence rate and a greater 5-year cancer-specific mortality rate (15). In another retrospective study that included 1,029 patients from 10 Canadian institutions between 1990 and 2010, Mason et al. reported that pLND >20% was associated with decreased CSS, recurrence-free survival, and OS (20). Moreover, Raza and colleagues verified the prognostic value of 30% pLND as the cutoff value in node-positive UTUC patients, utilizing the National Cancer Database, and the result is positive (21). These findings reinforced the conclusion that pLND could be used for risk stratification and survival prediction for patients with node-positive UTUC.

So far, the standard cross-sectional imaging has limited the accuracy in predicting LNM directly or distinguishing individuals who are at high risk of LNM (22, 23). Given that a proper postoperative nodal status is essential for the adequate management of patients and in the selection of patients who may benefit from adjuvant systemic therapy administration, sufficient LNs should be removed if lymphadenectomy is performed based on adverse clinical features. Our study is not devoid of limitations. First, the SEER database provides lymph node counts, but without defining a specific anatomic pelvic lymph node dissection template. Therefore, it is not possible to investigate whether template lymphadenectomy could improve the survival outcomes. We also excluded patients with less than three RLNs to make the RLN yields reach the LN yield in previous studies which conducted template lymphadenectomy. However, higher lymph node yield does not always mean a larger extent or a higher quality of lymphadenectomy. Second, the schedule or regime of adjuvant chemotherapy which has a potential impact on survival outcomes was not reported in the data repository. Third, the surgical method, such as open or laparoscopic, and the approach for bladder cuff management were not included in the SEER, and these factors may affect RLN yields or survival outcomes (24). Fourth, no data about smoking status or renal function before or after the operation were offered. Moreover, there might be some bias which is difficult to avoid due to the nature of retrospective studies.

## Conclusion

In summary, our results indicate that RLN yield during RNU was not associated with survival outcomes in patients with node-positive UTUC. However, a higher pLND could serve as an independent predictor of poor oncological outcomes. Combining pLND with other clinicopathological parameters can confer a more accurate survival prediction for node-positive UTUC patients. Therefore, to guide the further management of patients after surgery and achieve a more powerful prognosis, sufficient nodes should be removed if lymphadenectomy is performed. High-quality trials are needed to confirm and validate our findings.

## Data Availability Statement

Publicly available datasets were analyzed in this study. This data can be found here: https://seer.cancer.gov/. The accession number is “12896-Nov2021”.

## Ethics Statement

The data of our study were derived from the SEER database. All procedures performed in the studies involving human participants were in accordance with the ethical standards of the institutional and national research committee and with the 1964 Helsinki Declaration and its later amendments or comparable ethical standards. The SEER Program collects data from population-based cancer registries with anonymous information. It is a publicly available database; thus, no ethical approval is required.

## Author Contributions

X-xG: formal analysis and writing—original draft. H-rX: writing—review and editing. S-gL: data curation. X-qZ: data curation and writing—review, ML: conceptualization. J-yW: conceptualization, funding acquisition, resource provision, and supervision. All authors contributed to the article and approved the submitted version.

## Funding

The study was supported by Beijing Municipal Science and Technology Project (Z201100005620007) and Beijing Hospital Clinical Research 121 Project (BJ-2020-171).

## Conflict of Interest

The authors declare that the research was conducted in the absence of any commercial or financial relationships that could be construed as a potential conflict of interest.

## Publisher’s Note

All claims expressed in this article are solely those of the authors and do not necessarily represent those of their affiliated organizations, or those of the publisher, the editors and the reviewers. Any product that may be evaluated in this article, or claim that may be made by its manufacturer, is not guaranteed or endorsed by the publisher.

## Supplementary Material

Supplementary Table 1 Univariable Cox regression analyses of variables for overall survival and cancer-specific survival outcomes.

Supplementary Table 2 Multivariable Cox regression analyses of RLNs and covariables for overall survival and cancer-specific survival outcomes.

Supplementary Table 3 Multivariable Cox regression analyses of pLNs and covariables for overall survival and cancer-specific survival outcomes.
