# Supplementary material for: The Value of Lymph Node Dissection in Patients With Node-Positive Upper Urinary Tract Urothelial Cancer: A Retrospective Cohort Study
Source: Front Oncol. 2022 Jun 16;12:889144. doi: 10.3389/fonc.2022.889144 (PMC9245452; doi:10.3389/fonc.2022.889144)
Supplement: Supplementary Table 1 — Univariable Cox regression analyses of variables for overall survival and cancer-specific survival outcomes. [file DataSheet_1.docx]

|  | **CSS** | | **OS** | |
| --- | --- | --- | --- | --- |
|  | **HR (95% CI)** | **P-value** | **HR (95% CI)** | **P value** |
| **Age** | 1.01(1.00-1.02) | 0.075 | 1.00(0.99-1.01) | 0.149 |
| **Sex** |  |  |  |  |
| Male | ref. |  | ref. |  |
| Female | 1.07(0.95-1.72) | 0.274 | 0.82(0.47-1.08) | 0.163 |
| **Race** |  |  |  |  |
| Caucasian |  |  | ref. |  |
| African | 0.69(0.42-1.58) | 0.350 | 0.39(0.14-1.11) | 0.129 |
| Other | 0.83(0.56-1.29) | 0.127 | 0.78(0.25-1.14) | 0.414 |
| **Tumour site** |  |  |  |  |
| Renal pelvic | ref. |  | ref. |  |
| Ureter | 0.65(0.43-0.98) | 0.048 | 0.71(0.29-1.21) | 0.235 |
| **Laterality** |  |  |  |  |
| Left | ref. |  | ref. |  |
| Right | 1.17(0.84-1.32) | 0.372 | 1.08(0.75-1.43) | 0.497 |
| **Tumour size** |  |  |  |  |
| < 2 cm | ref. |  | ref. |  |
| ≥ 2 cm | 2.31(1.81-3.29) | < 0.001 | 2.45(1.23-3.27) | 0.010 |
| **T stage** |  |  |  |  |
| T2 | ref. |  | ref. |  |
| T3 | 2.05(1.19-3.75) | 0.009 | 1.76(0.95-3.99) | 0.095 |
| T4 | 4.74(3.41-6.89) | < 0.001 | 4.05(3.56-8.34) | < 0.001 |
| **Pathological grade** |  |  |  |  |
| Low grade | ref. |  | ref. |  |
| High grade | 0.85(0.34-1.34) | 0.383 | 0.55(0.23-1.01) | 0.064 |
| **Adjuvant radiotherapy** |  |  |  |  |
| No | ref. |  | ref. |  |
| Yes | 1.23(0.45-2.88) | 0.485 | 0.95(0.74-1.46) | 0.461 |
| **Adjuvant chemotherapy** |  |  |  |  |
| No | ref. |  | ref. |  |
| Yes | 2.17(1.55-3.03) | < 0.001 | 3.87(2.35-5.14) | < 0.001 |
| **RLNs** | 1.01(1.00-1.01) | 0.718 | 1.01(1.00-1.02) | 0.624 |
| **pLNs** | 1.02(0.99-1.05) | 0.158 | 1.02(0.99-1.06) | 0.087 |
| **pLND** | 1.01(1.00-1.01) | 0.018 | 1.01(1.00-1.01) | 0.008 |

**Supplement table 1.** Univariable Cox regression analyses of variables for overall survival and cancer-specific survival outcomes

HR=hazard ratio, CI=confidential interval, CSS=cancer-specific survival, OS=overall survival, RLNs= removed lymph nodes, pLNs= positive lymph nodes, pLND = positive lymph node density

**Supplement table 2.** Multivariable Cox regression analyses of RLNs and covariables for overall survival and cancer-specific survival outcomes.

|  | **CSS** | | **OS** | |
| --- | --- | --- | --- | --- |
|  | **HR (95% CI)** | **P-value** | **HR (95% CI)** | **P value** |
| **Age** | 1.00(0.98-1.03) | 0.883 | 1.01(1.00-1.03) | 0.049 |
| **Sex** |  |  |  |  |
| Male | ref. |  | ref. |  |
| Female | 1.13(0.82-1.57) | 0.344 | 0.76(0.47-1.21) | 0.246 |
| **Race** |  |  |  |  |
| Caucasian | ref. |  | ref. |  |
| African | 0.78(0.38-1.60) | 0.289 | 0.48(0.17-1.33) | 0.077 |
| Other | 0.84(0.51-1.37) | 0.269 | 0.87(0.43-1.77) | 0.471 |
| **Tumour site** |  |  |  |  |
| Renal pelvic | ref. |  | ref. |  |
| Ureter | 0.67(0.45-1.00) | 0.061 | 0.62(0.34-1.13) | 0.148 |
| **Laterality** |  |  |  |  |
| Left | ref. |  | ref. |  |
| Right | 1.05(0.76-1.44) | 0.724 | 1.01(0.64-1.58) | 0.948 |
| **Tumour size** |  |  |  |  |
| < 2 cm | ref. |  | ref. |  |
| ≥2 cm | 2.55(1.18-5.55) | 0.034 | 2.54(1.23-4.11) | 0.011 |
| **T stage** |  |  |  |  |
| T2 | ref. |  | ref. |  |
| T3 | 2.08(1.13-3.82) | 0.008 | 1.81(0.78-4.17) | 0.109 |
| T4 | 3.84(2.04-7.24) | < 0.001 | 3.97(1.67-9.43) | 0.001 |
| **Pathological grade** |  |  |  |  |
| Low grade | ref. |  | ref. |  |
| High grade | 2.57(0.61-12.4) | 0.217 | 2.48(0.94-6.49) | 0.058 |
| **Adjuvant radiotherapy** |  |  |  |  |
| No | ref. |  | ref. |  |
| Yes | 0.95(0.54-1.67) | 0.756 | 1.21(0.50-2.91) | 0.720 |
| **Adjuvant chemotherapy** |  |  |  |  |
| No | ref. |  | ref. |  |
| Yes | 2.17(1.55-3.03) | < 0.001 | 3.61(2.22-5.86) | < 0.001 |
| **RLNs** | 1.01(1.00-1.01) | 0.914 | 1.01(1.00-1.02) | 0.875 |

HR=hazard ratio, CI=confidential interval, CSS=cancer-specific survival, OS=overall survival, RLNs= removed lymph nodes

**Supplement table 3.** Multivariable Cox regression analyses of pLNs and covariables for overall survival and cancer-specific survival outcomes.

|  | **CSS** | | **OS** | |
| --- | --- | --- | --- | --- |
|  | **HR (95% CI)** | **P-value** | **HR (95% CI)** | **P value** |
| **Age** | 1.00(0.98-1.03) | 0.760 | 1.01(1.01-1.03) | 0.047 |
| **Sex** |  |  |  |  |
| Male | ref. |  | ref. |  |
| Female | 1.14(0.82-1.58) | 0.447 | 0.76(0.47-1.23) | 0.264 |
| **Race** |  |  |  |  |
| Caucasian | ref. |  | ref. |  |
| African | 0.72(0.35-1.46) | 0.362 | 0.42(0.15-1.17) | 0.097 |
| Other | 0.78(0.48-1.26) | 0.305 | 0.79(0.39-1.58) | 0.498 |
| **Tumour site** |  |  |  |  |
| Renal pelvic | ref. |  | ref. |  |
| Ureter | 0.69(0.46-1.04) | 0.073 | 0.66(0.36-1.19) | 0.167 |
| **Laterality** |  |  |  |  |
| Left | ref. |  | ref. |  |
| Right | 1.04(0.75-1.43) | 0.821 | 0.95(0.61-1.50) | 0.839 |
| **Tumour size** |  |  |  |  |
| < 2cm | ref. |  | ref. |  |
| ≥2 cm | 2.26(1.05-4.87) | 0.038 | 2.56(1.21-4.29) | 0.012 |
| **T stage** |  |  |  |  |
| T2 | ref. |  | ref. |  |
| T3 | 2.17(1.18-3.98) | 0.012 | 1.84(0.8-4.24) | 0.153 |
| T4 | 4.13(2.2-7.76) | < 0.001 | 4.23(1.79-9.99) | 0.001 |
| **Pathological grade** |  |  |  |  |
| Low grade | ref. |  | ref. |  |
| High grade | 2.68(0.58-12.5) | 0.208 | 2.51(0.96-6.57) | 0.060 |
| **Adjuvant radiotherapy** |  |  |  |  |
| No | ref. |  | ref. |  |
| Yes | 0.94(0.51-1.58) | 0.704 | 1.11(0.46-2.68) | 0.810 |
| **Adjuvant chemotherapy** |  |  |  |  |
| No | ref. |  | ref. |  |
| Yes | 2.21(1.58-3.07) | < 0.001 | 3.6(2.22-5.83) | < 0.001 |
| **pLNs** | 1.02(0.99-1.05) | 0.224 | 1.03(0.99-1.08) | 0.125 |

HR=hazard ratio, CI=confidential interval, CSS=cancer-specific survival, OS=overall survival, pLNs= positive lymph nodes
